# Supplementary material for: The interactome of intact mitochondria by cross-linking mass spectrometry provides evidence for coexisting respiratory supercomplexes
Source: Mol Cell Proteomics. 2017 Dec 8;17(2):216–32. doi: 10.1074/mcp.RA117.000470 (PMC5795388; doi:10.1074/mcp.RA117.000470)
Supplement: Supplemental Data [file supp_RA117.000470_133922_0_supp_23978_fzffwf.zip › spectra_annotation/mito_spectra_annotation/197-2-7-1-8-1.pdf]

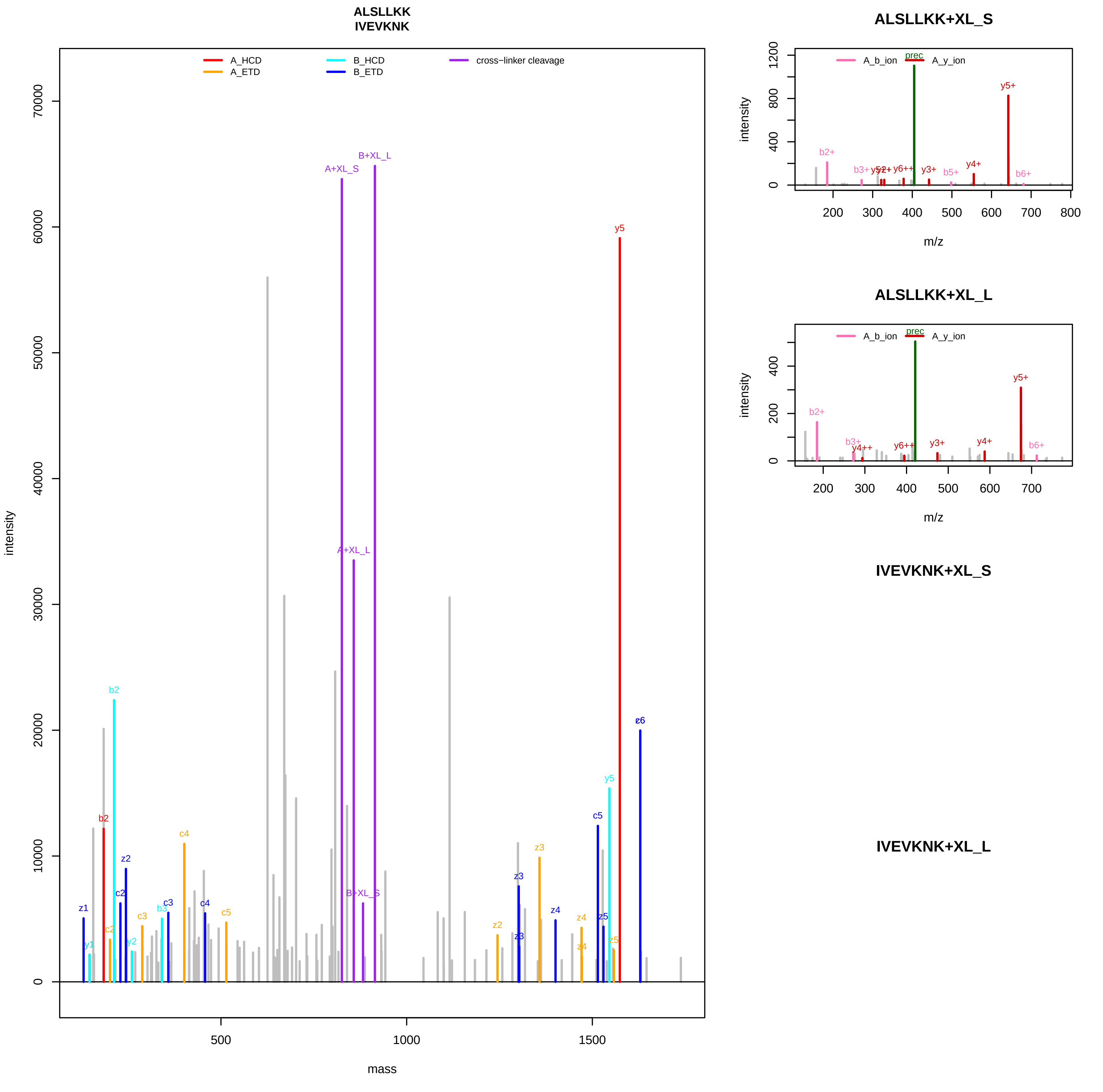
ALSLLKK
IVEVKNK
A_HCD
A_ETD
B_HCD
B_ETD
cross−linker cleavage
intensity
mass
A+XL_S
B+XL_L
A+XL_L
B+XL_S
y5
b2
z1
y1
c2
y2
c3
b3
c3
c4
c4
c5
z2
z3
z4
c5
z5
y5
z5
c6
ALSLLKK+XL_S
A_b_ion
prec
A_y_ion
b2+
b3+
y5+
y4+
b5+
b6+
y3+
y6++
m/z
ALSLLKK+XL_L
IVEVKNK+XL_S
IVEVKNK+XL_L
